# Supplementary material for: Tomographic detection of photon pairs produced from high-energy X-rays for the monitoring of radiotherapy dosing
Source: Nat Biomed Eng. 2022 Oct 24;7(3):323–34. doi: 10.1038/s41551-022-00953-8 (PMC10038801; doi:10.1038/s41551-022-00953-8)
Supplement: Supplementary file 1 — Supplementary figure and table. [file 41551_2022_953_MOESM1_ESM.pdf]

nature biomedical engineering

Article https://doi.org/10.1038/s41551-022-00953-8

Tomographic detection of photon pairs produced from high-energy X-rays for the monitoring of radiotherapy dosing

In the format provided by the authors and unedited

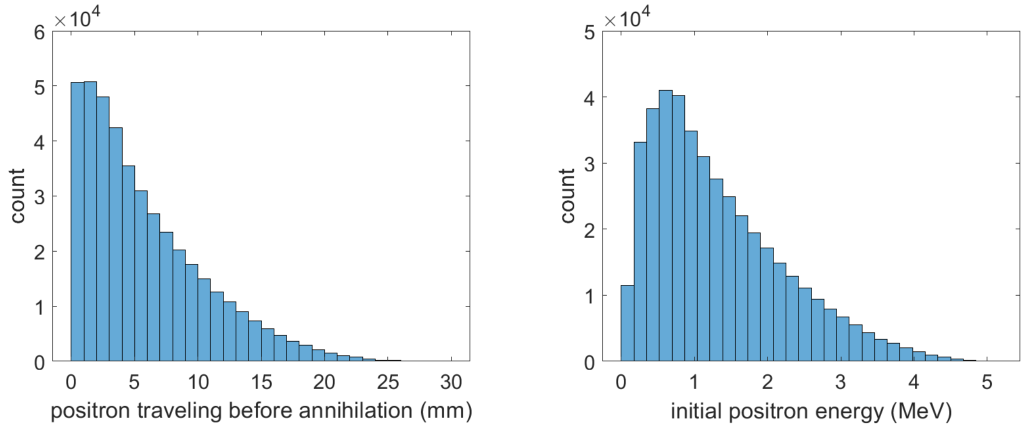

Supplementary Fig. 1 l A pair production positron will travel for a finite distance before annihilation. (a) The histogram of positron travelling distances before annihilation. (b). The histogram of initial positron energies from pair production.

Supplementary Table 1 l Material composition, density, and effective atomic number of the 10 inserts in the standard phantom, including air, lung inhale, lung exhale, adipose, breast, water, muscle, liver, trabecular bone, and dense bone.

Tissue	Composition (%)												ρ $(\mathrm{g\ cm^{-3}})$	Z_{eff}
	H	C	N	O	Na	Mg	P	S	Cl	K	Ca	Fe		
Air			70.0	30.0									0.001	7.3
Lung inhale	10.3	10.5	3.1	74.9	0.2		0.2	0.3	0.2	0.3			0.22	7.16
Lung exhale	10.3	10.5	3.1	74.9	0.2		0.2	0.3	0.2	0.3			0.51	7.16
Adipose	11.4	59.8	0.7	27.8	0.1			0.1	0.1				0.97	6.02
Breast	10.9	50.6	2.3	35.8	0.1		0.1	0.1	0.1				0.99	6.24
Water	11.2			88.8									1.00	7.22
Muscle	10.2	14.3	3.4	71.0	0.1		0.2	0.3	0.1	0.4			1.06	7.09
Liver	10.2	13.9	3.0	71.6	0.2		0.3	0.3	0.2	0.3			1.07	7.13
Trabecular	8.5	40.4	5.8	36.7	0.1	0.1	3.4	0.2	0.2	0.1	4.4	0.1	1.16	7.89
Dense Bone	5.6	23.5	5.0	43.4	0.1	0.1	7.2	0.3	0.1	0.1	14.6		1.58	10.48
